# Nanoscale Fe_3_O_4_ Electrocatalysts for Oxygen Reduction Reaction

**DOI:** 10.3390/molecules30081753

**Published:** 2025-04-14

**Authors:** Junjie Zhang, Jilong Wang, Yaoming Fu, Xing Peng, Maosong Xia, Weidong Peng, Yaowei Liang, Wuguo Wei

**Affiliations:** 1 Aerospace Vehicle Power Engineering, Institute of Aeronautical Engineering, Civil Aviation Flight University of China, Tianfu Campus, Chengdu 618000, China; wjl8655765580@126.com (J.W.); ymfo@163.com (Y.F.); fypx3688@163.com (X.P.); qq313674106@163.com (M.X.); pwdcafuc@163.com (W.P.); 2 Department of Mechanical & Industrial Engineering, University of Toronto, Toronto, ON M5S 2E8, Canada; liangyaowei612@gmail.com

**Keywords:** Fe_3_O_4_ electrocatalyst, ORR, hydrothermal synthesis, SDS, electrochemical stability

## Abstract

This study presents a straightforward hydrothermal synthesis approach to fabricate uniform and highly dispersed nanoscale Fe_3_O_4_ electrocatalysts for the oxygen reduction reaction (ORR). FeSO_4_·7H_2_O is used as the precursor, and sodium dodecyl sulfate (SDS) is incorporated as a dispersing agent to optimize particle size and dispersion. The SDS concentration plays a crucial role in controlling the particle size and distribution, with higher SDS concentrations resulting in smaller, well-dispersed particles (30–40 nm), compared to the agglomerated particles formed without SDS. The Fe_3_O_4_ catalyst demonstrates significant enhancement in ORR performance, with a half-wave potential of 0.091 V vs. Hg/HgO and a limiting diffusion current density of −5.50 mA cm^2^, surpassing the performance of agglomerated Fe_3_O_4_ and approaching that of state-of-the-art 20% Pt/C catalysts. Additionally, the Fe_3_O_4_ catalyst exhibits superior stability and resistance to methanol and CO poisoning, presenting a promising alternative to platinum-based catalysts for ORR applications. This work introduces an efficient approach for the synthesis of high-performance and evenly distributed Fe_3_O_4_ electrocatalysts, offering a new pathway for the development of metal oxide-based ORR catalysts with enhanced activity and durability.

## 1. Introduction

As traditional energy resources deplete and concerns about environmental degradation and CO_2_ emissions intensify, finding clean, sustainable, and eco-friendly alternative energy sources has become an urgent priority [1,2,3,4,5,6]. Zinc–air batteries are regarded as promising metal–air batteries because of their flat discharge voltage, high theoretical energy density, effective energy conversion, and silent operation [7,8,9,10,11,12]. One of the most critical factors affecting the energy conversion efficiency of zinc–air batteries is the slow kinetic process of the oxygen reduction reaction (ORR) at the cathode [13,14,15]. Currently, platinum (Pt) and its alloys are the most efficient ORR electrocatalysts; however, their widespread application is hindered by high cost, limited supply, instability, and potential toxicity [8,16,17,18]. Significant research efforts have been dedicated to developing cost-effective and efficient ORR electrocatalysts to replace Pt and its alloys. Promising alternatives include precious metal alloy-based materials [19], metal oxide-based materials [20,21], and heteroatom-doped carbon-based materials [22,23,24].

Iron (Fe) oxide-based materials have emerged as promising alternatives to Pt and its alloys due to their remarkable electrocatalytic performance, tolerance to methanol and CO, and long-term durability [25,26,27]. For example, Gao developed a Fe_3_O_4_ catalyst (NC@ Fe_3_O_4_-900-1.5) with a particle size of approximately 1 μm, exhibiting excellent ORR performance, including a limiting diffusion current density of 4.5 mA cm^−2^ and a half-wave potential of −0.05 V vs. Hg/HgO in a 0.1 M KOH solution [28]. Chen synthesized uniform Fe_3_O_4_ catalysts (Fe_3_O_4_/HCS-600) with an average particle size of approximately 170 nm, exhibiting a limiting diffusion current density of 3.7 mA cm^−2^ and a half-wave potential of −0.22 V vs. Ag/AgCl in alkaline electrolytes, along with enhanced stability during cycling and steady-state polarization tests [29]. Furthermore, Wang synthesized Fe_3_O_4_ catalysts (Fe-CNS-N) with particle sizes ranging from 150 to 200 nm. These catalysts exhibited remarkable resistance to methanol poisoning and demonstrated outstanding ORR performance [30]. Previous studies have elucidated that Fe oxide-based ORR electrocatalysts predominantly utilize active sites comprised of Fe^2+^ and Fe^3+^ ions, which modulate the electronic distribution, thereby enhancing oxygen molecule adsorption, reduction, and desorption rates [31]. However, the large size of Fe oxide limits its electrocatalytic activity. The large size and agglomeration restrict the exposure of active sites, and the irregular morphology further reduces the electrocatalytic activity. Consequently, achieving effective control over the morphology of Fe oxide to enable small, uniform, nanoscale sizes and high dispersion has become a significant focus of current research.

Surfactants are important reagents that can control material morphology during hydrothermal synthesis, guiding the growth direction and shape of crystals by adsorbing on crystal faces or surfaces, thus achieving precise control over material size and morphology. In the study of Fe oxide, surfactants have been widely used to control its morphology and improve its electrocatalytic performance. For example, using cetyltrimethylammonium bromide and sodium dodecyl sulfate (SDS) surfactants, Chouchainef et al. successfully controlled the morphology of Fe_3_O_4_ particles, yielding sizes of 150–300 nm and 50–80 nm, respectively, and optimized their capacitance performance, demonstrating the relationship between capacitance performance and particle size and shape [32]. Wang used F127 surfactant to regulate the synthesis of morphology-uniform Fe_3_O_4_ spheres with a size of approximately 170 nm from Fe (NO_3_)_3_·9H_2_O [30]. Shen et al. successfully controlled the particle size of Fe_3_O_4_ particles between 60 and 70 nm using trisodium citrate trinitrate (TSCD) surfactant. In summary, surfactants can not only effectively control the size of Fe_3_O_4_ materials but also regulate their electrochemical properties [33]. However, the currently reported Fe_3_O_4_ particles still exhibit large sizes, agglomeration, and non-uniformity, indicating potential for further optimization.

This work aims to control the high dispersibility, small nanoscale, and uniform morphology of Fe_3_O_4_ particles during hydrothermal synthesis using the surfactant SDS and annealing to improve their electrocatalytic performance. By increasing the content of the surfactant SDS during the hydrothermal process, this work successfully reduced the size of Fe_3_O_4_ particles from 100 to 500 nm (S0-Fe_3_O_4_ without SDS) to 30–40 nm (S2-Fe_3_O_4_). When compared to agglomerated S0-Fe_3_O_4_ without SDS (−0.064 V and −3.68 mA cm^2^), the half-wave potential (0.091 V vs. Hg/HgO) and limiting diffusion current density (−5.50 mA cm^2^) of S2-Fe_3_O_4_ improve by about 155 mV and 1.82 mA cm^2^, respectively, and even approach the performance of 20% Pt/C (0.092 V and −5.51 mA cm^2^). Furthermore, as compared to 20% Pt/C, S2-Fe_3_O_4_ exhibits better stability and resistance to CO poisoning and methanol. By using a zinc plate as the anode and the S2-Fe_3_O_4_ catalyst as the cathode, a zinc–air battery is created that can effectively light an LED with a voltage of 1.33 V and a current density of 44.5 mA. This work paves a novel route for synthesizing highly dispersed, small nanoscale, and uniform metal oxide particles as high-performance ORR catalysts by introducing an SDS-assisted approach that effectively addresses the issues of metal oxide aggregation and large particle size.

## 2. Experimental

### 2.1. Materials and Synthesis Methodology

**Figure 1:**
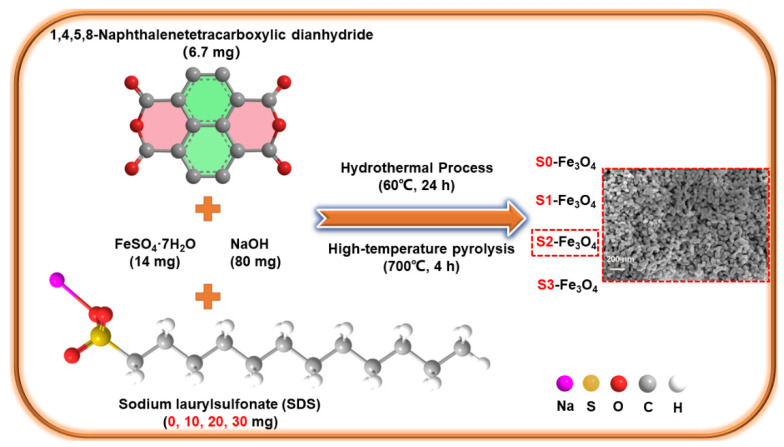
Preparation of a series of S0-Fe_3_O_4_~S3-Fe_3_O_4_ catalysts.

The following steps were followed according to the previously reported literature [34]. Dissolve 6.7 mg of 1,4,5,8-naphthalenetetracarboxylic dianhydride (NTCDA) and 80 mg of NaOH in 200 mL of high-purity water to form a homogeneous solution. The chemicals NTCDA and NaOH were supplied by Guanghua Sci-Tech Co., Ltd., Shantou, China, To the solution, add 100 mL of a water-based solution comprising 14 mg of FeSO_4_·7H_2_O and 0, 10, 20, and 30 mg of sodium laurylsulfonate (SDS), and mix for half an hour. SDS and FeSO_4_ were procured from Zhonglian Chemical, Jinan, China. The purity of FeSO_4_ reaches 95%. Then, conduct a 24 h hydrothermal reaction at 60 °C. The precipitate is obtained after centrifugation. The synthetic sample is heated in an N_2_ environment for 4 h at 700 °C. The temperature increase rate applied in this process is 10 °C min^−1^. The final products are cooled to room temperature and are identified as S0-Fe_3_O_4_, S1-Fe_3_O_4_, S2-Fe_3_O_4_, and S3-Fe_3_O_4_. The above-mentioned samples are subjected to the precipitation and centrifugation processes. After the precipitation and centrifugation operations are completed, the resulting precipitate is washed three times with deionized water to ensure that impurities are thoroughly removed. To improve the dispersibility of the precipitate, it is subsequently placed in a mortar and ground. The above details are shown in Figure 1. The intermediate product S2-Fe_3_O_4_-B forms during hydrothermal reaction, preceding annealing.

### 2.2. Characterization

X-ray diffraction (XRD) was performed using a D/MAX-Ultima+ diffractometer with Co Kα radiation, Rigaku Corporation, located in Tokyo, Japan. N_2_ adsorption–desorption measurements were conducted at 77 K using a WBL-810 device manufactured by Wenzhou Ruixin Instrument Co., Ltd., located in Wenzhou, China. The microstructural morphology of the produced samples was examined with a Supra-55 sapphire fitted FE–SEM, and the acceleration voltage of the FE–SEM was 5 kV, Carl Zeiss AG, Oberkochen, Germany. X-ray photoelectron spectroscopy (XPS) characterization was carried out using a VG Micro-tech ESCA 2000 apparatus with monochromatic Al Kα X-rays, Thermo Fisher Scientific, Waltham, MA, USA. High-resolution TEM (HR-TEM) images were acquired using a JEM-2100 microscope by JEOL Ltd., Tokyo, Japan. The Fourier Transform Infrared Spectroscopy (FTIR) measurement was carried out using the Prestige21, an instrument manufactured by Shimadzu, Kyoto, Japan. This instrument has a measurement range spanning from 500 to 4500 cm^−1^. Nano measurement 1.2.5 software was employed to statistically analyze the particle size distribution of Fe_3_O_4_.

### 2.3. Electrochemical Measurements

The electrochemical studies were carried out using a Corrtest CS310 M electrochemical station, Wuhan Corrtest Instruments Corp. Ltd., Wuhan, China. A graphite rod was used as the counter electrode and a Hg/HgO electrode as the reference electrode, with a glass carbon electrode acting as the working electrode. The diameter of the glassy carbon electrode was 0.561 cm. Following a combination of 10 mg sample, 5 mL isopropanol, and 20 µL 5% Nafion, the mixture underwent a 20 min ultrasonography treatment. Experiments using LSV (linear sweep voltammetry) and CV (cyclic voltammetry) were conducted at scan speeds of 10 and 20 mV s^−1^, respectively. At rotating speeds between 400, 900, 1600, and 2500 rpm, LSV curves were recorded in the KOH solution (0.1 M).

To obtain the transferred electron number (N), the Koutechy-Levich (K-L) curve’s slope is calculated using Equations (1) and (2) [35].

(1) 1J=1JL+1JK=1Bω12+1JK

(2) B=0.2nFC0D23ν−16

The current density measured during the electrochemical experiments is denoted as J. Among them, J_k_ represents the kinetic current density, and J_L_ represents the limiting current density. The unit of the above-mentioned current densities (J, J_k_, and J_L_) is mA cm^−2^. n represents the number of transferred electrons. B, with the unit of C cm s^−1/2^, represents the Levich slope. The rotation rate of the electrode is denoted as ω, which is 400, 900, 1600, or 2500 rpm, respectively. F is the Faraday constant (F = 96,486 C mol^−1^). D is the diffusion coefficient of oxygen (1.9 × 10^−5^ mol cm^−3^ s^−1^). C_0_ is the oxygen bulk concentration (1.2 × 10^−3^ mol cm^−3^). v is the kinetic viscosity (0.01 cm^−2^ s^−1^).

Measurements with a rotating ring-disk electrode (RRDE) were conducted. The area of the disk is 0.247 cm^2^, and that of the Pt ring is 0.06 cm^2^. The percentage productivity of peroxide (% HO_2_) is calculated using the equation:

(3) $$\%\;HO_{2}=200*\frac{Ir/N}{Id+Ir/N}$$

Id and Ir are the disk and ring electrode current density measured at 1600 rpm, respectively. The Pt ring electrode is polarized at −0.40 V, which can transform H_2_O_2_ into H_2_O. N is the collection efficiency of the ring electrode (N = 0.37).

Chronoamperometry (CA) tests are also conducted at −0.4 V to demonstrate the durability of the as-synthesized electrocatalyst in O_2_-saturated alkaline electrolyte. Accelerating Durability Test (ADT) evaluates catalyst durability via a 10,000 cycle CV test at 20 mV s^−1^.

### 2.4. DFT Calculations

The adsorption free energy of descriptors (O*, OH*, and OOH*) are calculated using the CASTEP module from Material Studio 2020 software. GGA-PBE is the function that is selected. The pseudopotential and energy cutoff are ultrasoft and 500 eV. The solvation model was COMSO, and the dielectric constant is 78.5 (H_2_O). In this work, a model of Fe_3_O_4_ is established, and the adsorption energies of the intermediate states OOH*, OH*, and O* are analyzed based on the (311) crystal plane. It is mainly attributed to the fact that (311) is the main crystal plane, which is characterized by XRD. Pt (111), as the main crystal plane, adsorbs the intermediate products according to the literature [36]. Vacuum thickness is 20.0 Å. The energy convergence for the self-consistent iteration of crystal structure relaxation and static energy calculations is 1.0 × 10^−5^ eV per atom for both. Force, displacement, and stress of the atomic relaxation standard are set to 0.01 eV Å, 5.0 × 10^−4^ Å, and 0.02 GPa, respectively. The adsorption free energy (G) is calculated using the equation, G = E + ZPE TS, where E, ZPE, T, and S are electric energy, zero-point energy, temperature, and entropy, respectively. The combining mechanism in an alkaline solution is called the ORR process, and it consists of the following four phases. A sign of ORR activity may be found in the step reactions’ maximum energy barrier. A high reaction energy barrier indicates low ORR activity.

O_2_+* + H_2_O + e^−^ ➝ OOH* + OH (4)

OOH* + e^−^ ➝ O* + OH^−^ (5)

O* + H_2_O + e^−^ ➝ OH* + OH^−^ (6)

OH* + e^−^ ➝ OH^−^+* (7)

The adsorption condition on the electrode surface is represented by the star symbol (*) in Equations (4) through (7). The Norskov technique is the foundation for the energy barrier computation.

### 2.5. Assembly of the Zinc–Air Battery

**Figure 2:**
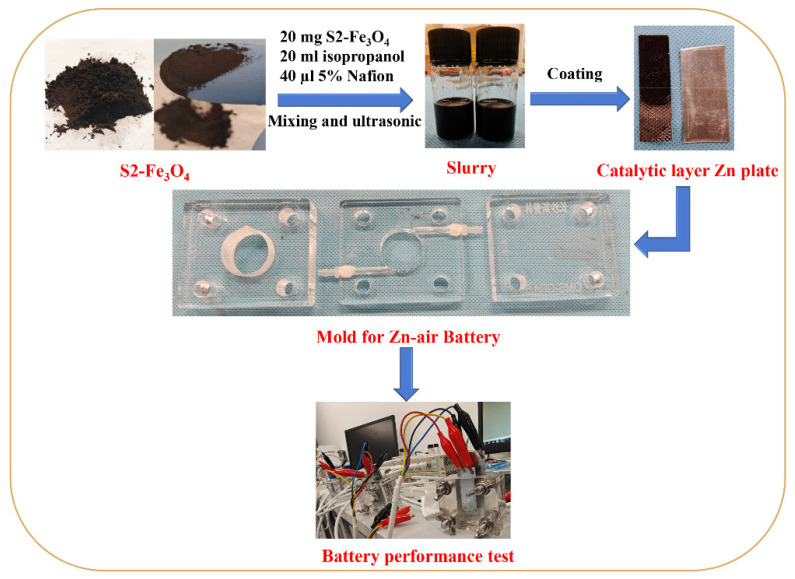
Flowchart depicting the experimental assembly process of the Zn–air battery.

Mixed together were 20 mg of Fe_3_O_4_ samples, 20 mL of isopropanol, and 20 µL of 5% Nafion. The mixture was then subjected to an ultrasonography treatment for 20 min, and thus the catalyst slurry was prepared. The prepared catalyst slurry was evenly and uniformly coated onto the cathode carbon paper (1.5 × 5.0 cm) by using the pipette and a scraper. The coating volume was 20 mL, which means 20 mg of the catalyst. Subsequently, it was left to volatilize at room temperature for 24 h to ensure that the catalyst adhered to the surface of the support. The details are shown in Figure 2.

## 3. Results and Discussions

### 3.1. Structure Analysis

**Figure 3:**
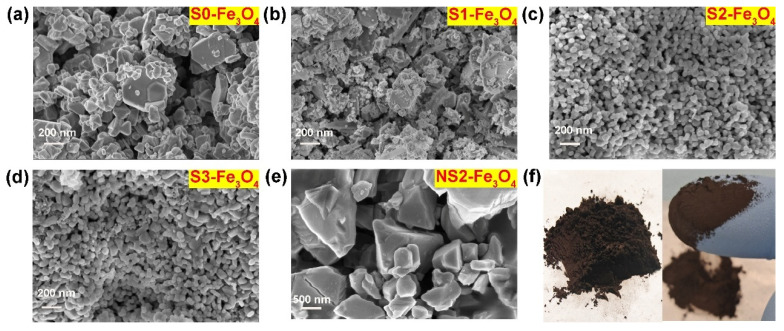
(**a**–**e**) SEM images of a series of S0-Fe_3_O_4_~S3-Fe_3_O_4_ and NS2-Fe_3_O_4_ catalysts; (**f**) Optical photograph of S2-Fe₃O₄ powder.

SEM images provide direct observation of the catalyst morphology. In the absence of SDS, the S0-Fe_3_O_4_ catalyst exhibits a bright block-like morphology with irregular and heterogeneous features. The particle size ranges from approximately 100 to 500 nm, with the main distribution centered around 100 to 200 nm, as shown in Figure 3a. However, upon the addition of 10 mg of SDS, significant changes in the morphology of the catalyst (S1-Fe_3_O_4_) are observed in Figure 3b. The block-like structures gradually fragment, leaving behind residue particles with a size range of approximately 0 to 400 nm, predominantly distributed around 50 to 200 nm, indicating a transitional state. With a further increase in SDS content (20 mg), the S2-Fe_3_O_4_ particles exhibit highly dispersed, uniform, and regular spherical-like morphology, ranging in size from 30 to 40 nm, with a dominant distribution around 35 nm, as shown in Figure 3c. A subsequent increase in SDS content (30 mg) did not cause a significant change in the morphology of the catalyst (S3-Fe_3_O_4_), suggesting the ineffectiveness of excessive SDS in controlling the catalyst morphology, as shown in Figure 3d.

The molar mass of FeSO_4_ is approximately 151 g mol^−1^. The mass of FeSO_4_ is 14 mg (equivalent to 0.014 g), and the amount of substance of Fe in FeSO_4_ is 9.27 × 10^−5^ mol. The molar mass of SDS is 288 g mol^−1^, and the molar amount of 0, 10, 20, and 30 mg is 0, 3.47 × 10^−5^, 6.94 × 10^−5^, and 1.04 × 10^−4^ mol, respectively. After calculation, the molar ratio of Fe to SDS is 0, 2.67:1, 1.34:1, and 0.89:1, respectively. As the molar ratio of Fe to SDS decreases (from 2.67:1 to 0.89:1), indicating an increase in the SDS content, more SDS surfactant molecules are likely to surround Fe ions. This phenomenon effectively inhibits the agglomeration of Fe ions, thereby facilitating the formation of Fe oxide particles with dispersibility and small particle size. The above indicates that an appropriate amount of SDS facilitates the formation of nanoparticles. The observed nanoparticle morphology can be mainly attributed to the properties of SDS, which is an anionic surfactant. SDS possesses a sulfate group, which is hydrophilic, and an alkyl group, which is hydrophobic. In a solution, the sulfate group exists as a negatively charged anion, leading to the interaction with transition metal ions. Consequently, electrostatic adsorption occurs between SDS and Fe ions in the solution. This electrostatic adsorption prevents the agglomeration of Fe_3_O_4_ particles, leading to the formation of uniformly dispersed nanoparticles [37,38]. The sample S2-Fe_3_O_4_ shows high dispersion, small nanoscale, and uniform characteristics when the SDS level is 20 mg, according to the data above. This morphology overcomes the drawbacks of previously reported large-sized catalysts, agglomeration, and non-uniformity. Samples without NTCDA (named NS2-Fe_3_O_4_) were prepared to demonstrate the role of NTCDA in Figure 3e. Observations indicate that the sizes of these samples range from 0.5 to 2 μm and their shapes are irregular, suggesting that NTCDA can indeed effectively reduce the size of the samples. In Figure 3f, the optical photograph of S2-Fe_3_O_4_ reveals it to be in the form of a black powder. When rubbed between the fingers, the particles of S2-Fe_3_O_4_ are found to be extremely fine, indicating that S2-Fe_3_O_4_ exists in powder form. In Figure 3c,f, the Fe_3_O_4_ nanoparticles are dispersed with gaps between each other, which may form a pore structure.

**Figure 4:**
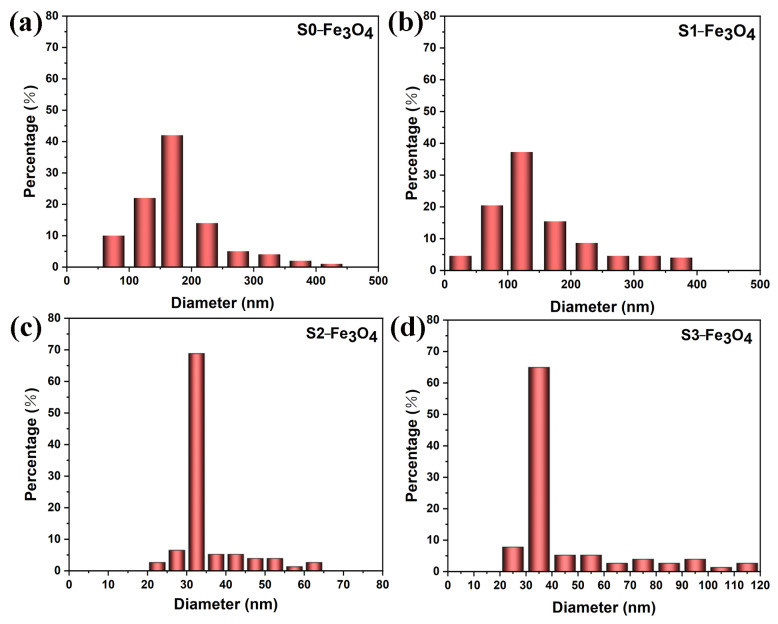
(**a**–**d**) Particle size distribution of a series of S0-Fe_3_O_4_~S3-Fe_3_O_4_ catalysts.

The particle size analysis results are depicted in Figure 4a–d. The number of particles is 507, 569, 1276, and 982 from S0-Fe_3_O_4_ to S3-Fe_3_O_4_, respectively, which was obtained with the nano measurement software. The S0-Fe_3_O_4_ sample, which does not contain SDS, displays a dominant particle size distribution ranging from 150 to 200 nm, constituting approximately 42% of the total. Upon the addition of 10 mg of SDS, the S1-Fe_3_O_4_ sample maintains a primary particle size distribution in the 100–150 nm range, but the percentage decreases to approximately 38%. These findings indicate that the addition of SDS results in a trend of particle size reduction, demonstrating the effective role of SDS in decreasing particle size.

**Figure 5:**
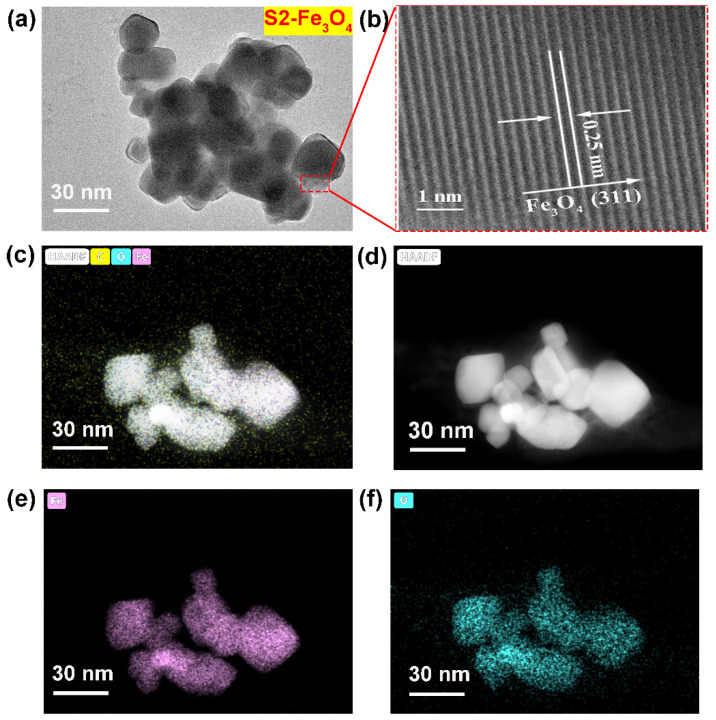
(**a**,**b**) TEM and HR-TEM images; (**c**–**f**) Element mapping and HAADF images of S2-Fe_3_O_4_-based catalysts.

For the S2-Fe_3_O_4_ sample, a further increase in SDS mass to 20 mg results in a considerable drop in particle size, with most of the particle size distribution, roughly 68%, concentrated in the 30 to 40 nm region. This observation further supports the notion that SDS facilitates particle size reduction. When the mass of SDS is further increased to 30 mg, the S3-Fe_3_O_4_ sample shows a particle-size distribution similar to that of S2-Fe_3_O_4_. Specifically, the proportion within the 30 to 40 nm range is approximately 68%. The above further emphasizes that S2-Fe_3_O_4_ is more suitable as an electrocatalyst. Based on SEM observations and particle size analysis, it is evident that the S2-Fe_3_O_4_ sample with the addition of 20 mg SDS displays highly dispersed, uniform, and small-sized spherical particles. This optimized morphology and size are advantageous for enhancing electrocatalytic performance, demonstrating its high catalytic potential. Figure 5a shows the TEM image of S2-Fe_3_O_4_. The results show that the S2-Fe_3_O_4_ sample has particles that are between 30 and 40 nm in size, which is in line with the previously stated SEM findings. Figure 5b shows a high-resolution transmission electron microscopy (HR-TEM) image, which reveals a lattice spacing of 0.25 nm. According to the literature [39,40], the (311) crystal plane is the main crystal plane in Fe_3_O_4_, with a lattice spacing of 0.25 nm, indicating the possibility that the material is Fe_3_O_4_.

**Figure 6:**
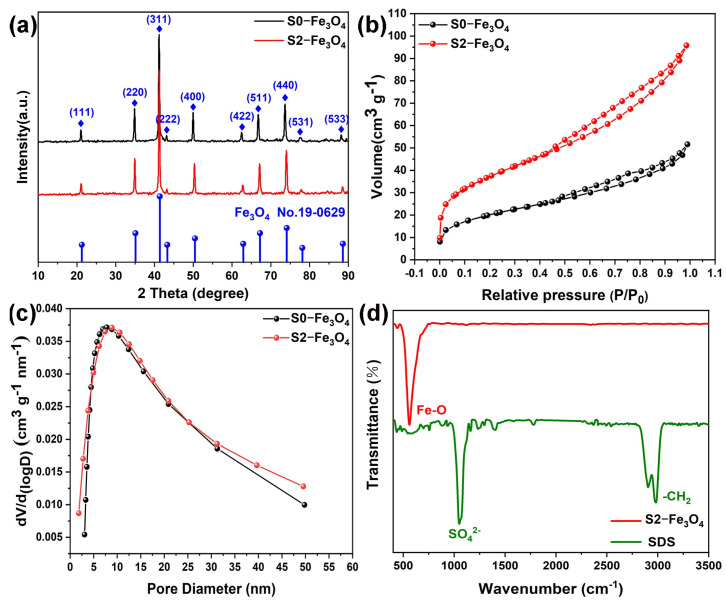
(**a**,**b**) XRD patterns and N_2_ adsorption–desorption curves; (**c**) Pore distribution of S0-Fe_3_O_4_ and S2-Fe_3_O_4_ catalysts; (**d**) FTIR spectrum of S2-Fe_3_O_4_ and SDS.

Furthermore, Figure 5c,d depict the results of High Angle Annular Dark Field (HAADF) imaging, confirming the particle size to be around 30 nm. Fe and O elements are present, according to elemental analysis displayed in Figure 5e,f, which is consistent with the findings of the SEM mapping. The function and purpose of element mapping is to confirm the presence of Fe and O elements, indicating that the substance is likely an iron oxide compound. Figure 6a shows the XRD spectra of S0-Fe_3_O_4_ and S2-Fe_3_O_4_, indicating that both samples consist of Fe_3_O_4_ (Card no. 19-0629) phase [41]. The similarity in the intensity of the diffraction peaks suggests that the addition of SDS does not affect the crystalline phase of the material. Consistent with the above HR-TEM observations, it was noted that the (311) crystal plane is the primary crystal plane of the sample.

Figure 6b illustrates the N_2_ adsorption–desorption isotherms of S0-Fe_3_O_4_ and S2-Fe_3_O_4_. They both display a type IV adsorption isotherm with a noticeable hysteresis loop in the high and medium-pressure areas, indicating the presence of mesoporous structures [42,43,44]. In addition, it is found that the intensity of the N₂ adsorption–desorption curve of S2-Fe_3_O_4_ is higher than that of S0-Fe_3_O_4_, indicating that the specific surface area of S2-Fe_3_O_4_ is larger than that of S0-Fe_3_O_4_. After calculation, it is found that the specific surface area of S2-Fe_3_O_4_ is 327 m^2^ g^−1^, which is higher than that of S0-Fe_3_O_4_ (51 m^2^ g^−1^). In Figure 6c, both S0-Fe_3_O_4_ and S2-Fe_3_O_4_ feature mesoporous pore size distributions. The pores range from 0 to 50 nm, and the two samples exhibit comparable pore volumes, which is consistent with the result of the N2 adsorption–desorption isotherm curve. FTIR tests of sample S2-Fe_3_O_4_ and SDS were conducted, and the results are shown in Figure 6d. SDS has stretching vibration peaks of CH_2_ at 2920 cm^−1^ and 2850 cm^−1^, as well as characteristic peaks of sulfate groups at 1250 cm^−1^ [45]. S2-Fe_3_O_4_ only shows an absorption peak of the Fe-O bond at 550 cm^−1^ [46] and no other absorption peaks are observed, which indicates that S2-Fe_3_O_4_ does not contain SDS. FTIR spectroscopy tests are carried out on the powders of samples S2-Fe_3_O_4_-B, S2-Fe_3_O_4_, and NTCDA, as shown in Figure S2. It is observed that S2-Fe_3_O_4_ has an absorption peak of Fe-O stretching vibration at 500 cm^−1^. NTCDA has an absorption peak of C = O stretching vibration at 1700 cm^−1^, and there will be an absorption peak of O-H bond stretching vibration at around 2500 cm^−1^ [47]. It is worth noting that S2-Fe_3_O_4_-B has absorption peaks at 500, 1700, and 2500 cm^−1^ simultaneously, which further indicates that it is a complex of Fe_3_O_4_ and NTCDA. The initial solution is evaporated and concentrated to increase the concentration of the complex for convenient detection by infrared spectroscopy. The sample is named S2-Fe_3_O_4_-B-1. It is observed that it had absorption peaks at 500, 1700, and 2500 cm^−1^ simultaneously in Figure S2, which further indicated the presence of Fe (III) or Fe (II) complexes of NTDA.

**Figure 7:**
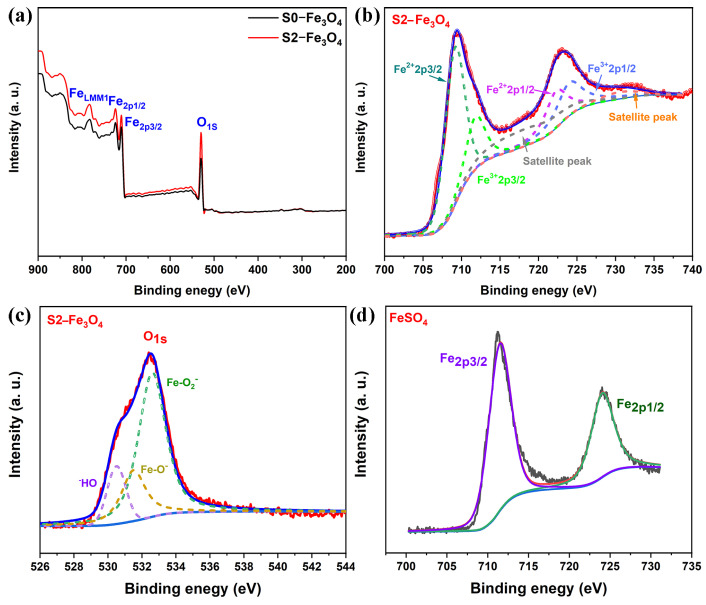
(**a**) Full scan XPS spectrum of S0-Fe_3_O_4_ and S2-Fe_3_O_4_; (**b**,**c**) High-resolution Fe and O of S2-Fe_3_O_4_; (**d**) High-resolution Fe of FeSO_4_.

XPS measurement enables the analysis of Fe atom types and their respective contents. As shown in Figure 7a, Fe and O element peaks are clearly observed. Furthermore, Fe2p_1/2_ and Fe2p_3/2_ peaks in S2-Fe_3_O_4_ exhibit stronger intensities compared to those of S0-Fe_3_O_4_, suggesting an elevated Fe content in S2-Fe_3_O_4_. Two peaks at 709.73 and 723.01 eV are the result of Fe2p_3/2_ and Fe2p_1/2_, respectively. Fe_2p3/2_ peaks can be deconvolved into Fe^3+^ and Fe^2+^ at 711.88 and 709.73 eV, while Fe_2p1/2_ peaks deconvolved into Fe^3+^ and Fe^2+^ at 725.04 and 723.01 eV in Figure 7b. Two satellite peaks are at 718.15 and 730.54 eV [48]. In Figure 7c, high-resolution of O1s is divided into Fe-O_2_^−^ (532 eV), Fe-O^−^ (531 eV), and OH^−^ (530 eV) [49,50]. Thereinto, OH- may originate from H_2_O. To verify whether Fe^2+^ in FeSO_4_ had been oxidized to Fe^3+^, XPS testing was conducted. As presented in Figure 7d, according to the literature [51], the Fe^3+^ states corresponding to 711 and 724 eV in the high-resolution spectrum of Fe are attributed to Fe^3+^ (FeOOH and Fe(OH)_3_). Therefore, the data indicate that the iron contained in the starting materials used for the synthesis exists in an oxidized state. In the hydrothermal reaction system [52,53], FeSO_4_ undergoes hydrolysis first, that is, Fe^2+^ + 2H_2_O→Fe (OH)_2_ + 2H^+^. In the presence of dissolved oxygen, an oxidation reaction takes place as follows: 4Fe (OH)_2_ + O_2_ + 2H_2_O → 4Fe (OH)_3_. Thereinto, Fe (OH)_3_ is not stable and will decompose, that is, 2Fe (OH)_3_ →Fe_2_O_3_ + 3H_2_O. Reactions occur among Fe (OH)_2_, Fe_2_O_3_, and incompletely decomposed Fe (OH)_3_, ultimately resulting in the formation of Fe_3_O_4_. The overall reaction can be approximately expressed as 6Fe (OH)_2_ + O_2_→2Fe_3_O_4_ + 6H_2_O. Based on the above physical characterization results, a series of Fe_3_O_4_ is successfully synthesized. The addition of SDS can effectively reduce particle size, enhance dispersion, increase Fe content, and improve specific surface area and pore density without altering the crystal lattice of the particles. This indicates that SDS can indeed help regulate the particle size, dispersivity, and uniformity of Fe_3_O_4_, which may further optimize its electrocatalytic performance.

### 3.2. Electrochemical Characterization

**Figure 8:**
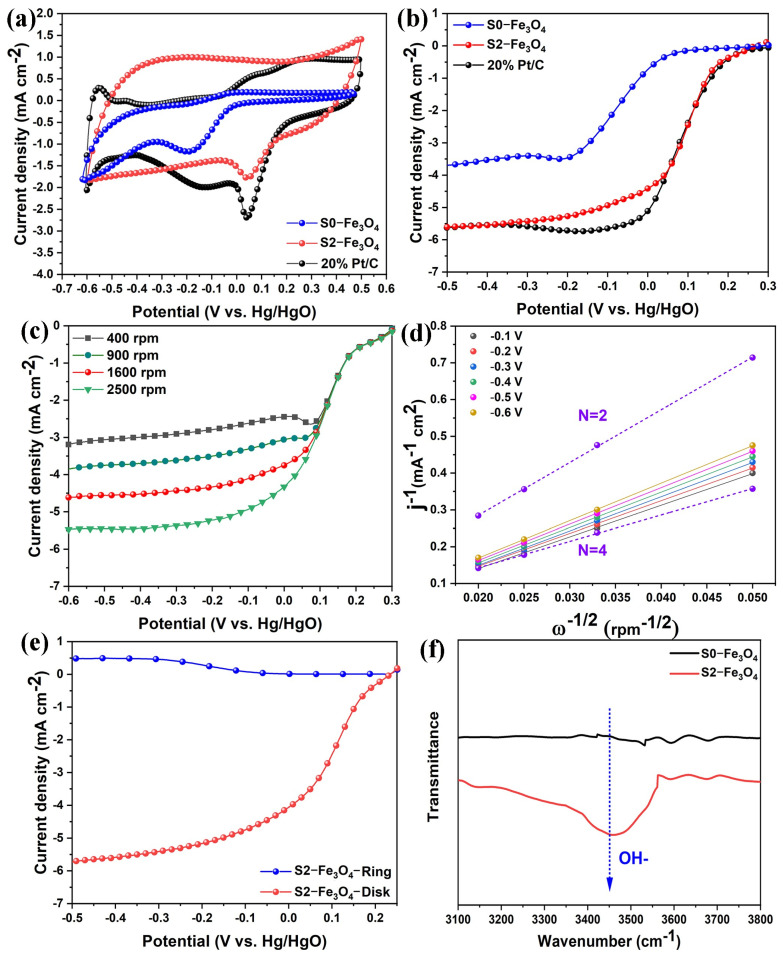
(**a**,**b**) CV, LSV curves of S0-Fe_3_O_4_, S2-Fe_3_O_4_ and 20% Pt/C; (**c**) RDE curves with 400, 900, 1600, and 2500 rpm; (**d**) K-L plots; (**e**) RRDE test of S2-Fe_3_O_4_; (**f**) FTIR spectra of the local OH⁻ regions of S0-Fe_3_O_4_ and S2-Fe_3_O_4_.

S0-Fe_3_O_4_, S2-Fe_3_O_4_, and 20% Pt/C ORR activities are measured in 0.1 M KOH solution using LSV and CV tests. Figure 8a shows that in 0.1 M KOH solution, ORR activity is present in S0-Fe_3_O_4_, S2-Fe_3_O_4_, and 20% Pt/C. Among them, the peak potential (0.040 V vs. Hg/HgO) of S2-Fe_3_O_4_ is enhanced about +25 mV compared with that of S0-Fe_3_O_4_ (−0.184 V), approaching 20% Pt/C (0.041 V). As shown in Figure 8b, this work measured LSV curves (@1600 rpm) in 0.1M KOH solution. For S2-Fe_3_O_4_, the potential range from 0.3 to 0.2 V is the ORR kinetic-controlled region, within which no faradaic current is generated. The potential range from 0.2 to 0 V is the mixed control region, encompassing the kinetic control region of the ORR and the oxygen transport control region, in which the Faraday current is produced. The potential range from 0 to −0.5 V is the oxygen transport control region. In the above region, the current reaches its maximum value, and the transport rate of oxygen is the crucial factor controlling the current density. Half-wave potential (0.091 V vs. Hg/HgO) and limiting diffusion current density (−5.50 mA cm^2^@ −0.5 V) of S2-Fe_3_O_4_ improve by approximately 155 mV and 1.82 mA cm^2^ compared to agglomerated S0-Fe_3_O_4_ without SDS (−0.064 V and −3.68 mA cm^2^), even approaching the performance of 20% Pt/C (0.092 V and −5.51 mA cm^2^). According to the above CV and LSV results, the electrocatalytic performance of S2-Fe_3_O_4_ is significantly improved after SDS addition, including peak potential, onset, half-wave potential, and limiting diffusion current density. S2-Fe_3_O_4_ even has a limiting diffusion current that is particularly near 20% Pt/C. On the contrary, the electrocatalytic performance of the S0-Fe_3_O_4_ without SDS was weaker than that of S2-Fe_3_O_4_. This may be attributed to the nanosize reduction of S2-Fe_3_O_4_, which can expose more active sites (Fe element), and thus improves the electrocatalytic performance, which is consistent with the above physical characterization results. On the contrary, the electrocatalytic performance of S0-Fe_3_O_4_ without SDS is weaker than that of S2-Fe_3_O_4_. This may be attributed to the small nanoscale and high dispersion of S2-Fe_3_O_4_, which can expose more active sites (Fe element), thus improving the electrocatalytic performance. This is consistent with the above physical characterization results.

LSV curves were acquired at 400, 900, 1600, and 2500 rpm to examine the ORR process of S2-Fe_3_O_4_, as shown in Figure 8c. As the rotation speed increased from 400 to 2500 rpm, the transport speed of oxygen was increased, thereby enhancing the limiting diffusion current density [54]. In Figure 8d, K-L graphs for S2-Fe_3_O_4_ are produced spanning the electrode potential range from −0.6 to −0.1 V, indicating initial-order reaction kinetics for O_2_ reduction and displaying high linearity. The ORR process may have a 4-electron channel as shown by the mean number of electrons transmitted (3.67) for S2-Fe_3_O_4_ [35]. The RRDE test was conducted to analyze the average number of transferred electrons for S2-Fe_3_O_4_ in Figure 8e. The average number of transferred electrons was found to be 3.6, aligning with the K-L results. The electrochemical testing process of S2-Fe_3_O_4_ may produce some by-products, such as H_2_O_2_, with an estimated yield of around 10%. The OH⁻ adsorption capacity of the material plays a crucial role in catalyzing ORR [55]. To compare the OH⁻ adsorption capabilities of S0-Fe_3_O_4_ and S2-Fe_3_O_4_, FTIR spectroscopy was employed. FTIR spectra show that the signal intensity of the OH⁻ species at ≈3450 cm^−1^ is the strongest for S2-Fe_3_O_4_ after ORR as compared with S0-Fe_3_O_4_ (Figure 8f), which strongly suggests that S2-Fe_3_O_4_ exhibits superior ORR performance.

**Figure 9:**
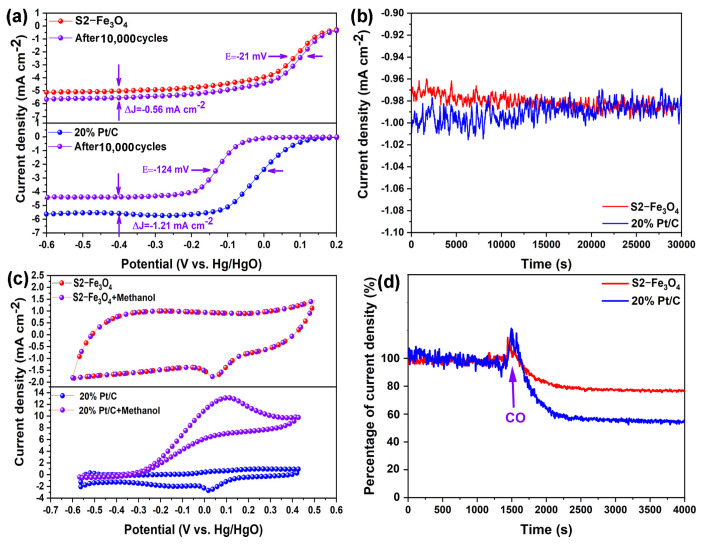
(**a**) LSV trends before and after 10,000 cycles; (**b**) Chronoamperometric responses; (**c**) CV curves in solution and with methanol; (**d**) The point of inflection revealed the addition of CO to the solution based on the chronoamperometric reflexes.

Accelerating durability test (ADT) was performed to evaluate catalyst durability and the results are shown in Figure 9a. After 10,000 cycles, the limited diffusion current density and half of a wave potential (@1600 rpm) of S2-Fe_3_O_4_ decreased by −0.56 mA cm^−2^ and −21 mV, respectively. For the 20% Pt/C, there is a more pronounced negative shift of −124 mV and −1.21 mA cm^−2^. Current density of S2-Fe_3_O_4_ remains stable at −0.98 mA cm^2^, while the current density of 20% Pt/C decreases from −1.0 to −0.98 mA cm^2^ during the same chronoamperometric response over 30,000 s in Figure 9b. One of the difficulties with direct methanol fuel cells is the crossing of methanol molecules over the membrane from the anode towards the cathode, which may contaminate the ORR catalyst. Therefore, it is crucial for ORR catalysts to exhibit high methanol tolerance to maintain optimal performance. As shown in Figure 9c, the activity of S2-Fe_3_O_4_ remains unaffected by the addition of 10 mL methanol, suggesting that it has a strong O_2_ selectivity and the capacity to counteract methanol’s crossover effects. Nevertheless, the ORR activity of 20% Pt/C is reduced by methanol oxidation processes, indicating that methanol interference is possible. It is challenging to eliminate CO gas as an impurity from fuel cells, which increases the risk of ORR catalyst poisoning. The plot in Figure 9d was used to assess the catalysts’ tolerance to CO. The current density of 20% Pt/C is reduced to around 58% when CO is added at 4000 s, and it has a lower resistance to CO poisoning than S2-Fe_3_O_4_ (about 77%). These results show that, in comparison to 20% Pt/C, S2-Fe_3_O_4_ has better durability and tolerance against methanol and CO.

**Figure 10:**
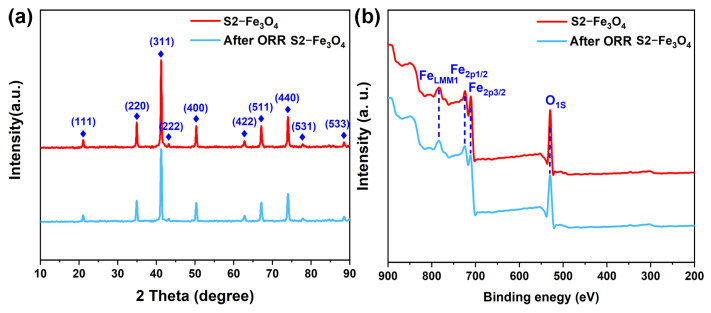
(**a**,**b**) XRD and XPS full-scan spectrum S2-Fe_3_O_4_ and after ORR S2-Fe_3_O_4_.

After 10,000 CV curves following ORR, neither the intensity of crystal planes nor the peak positions in the XRD patterns changed in Figure 10a. Additionally, the intensities of Fe and O elements in the XPS spectrum remained unaltered, as shown in Figure 10b. These results indicate that S2-Fe_3_O_4_ exhibits strong stability after the ORR process.

**Figure 11:**
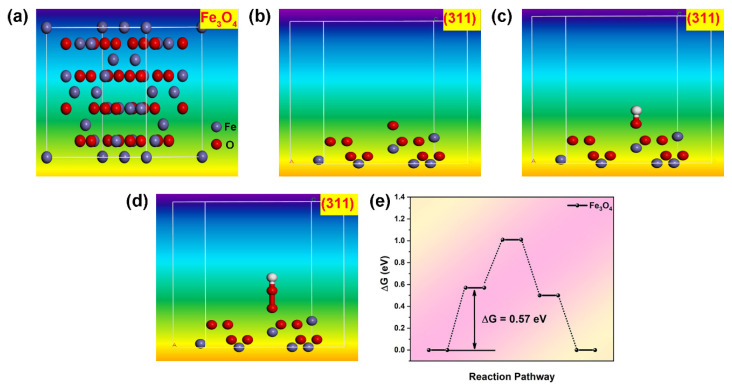
(**a**) Model image of Fe_3_O_4_; (**b**–**d**) O*, OH*, OOH* adsorption state on (311) of FeO; (**e**) Reaction step diagram of Fe_3_O_4_.

**Figure 12:**
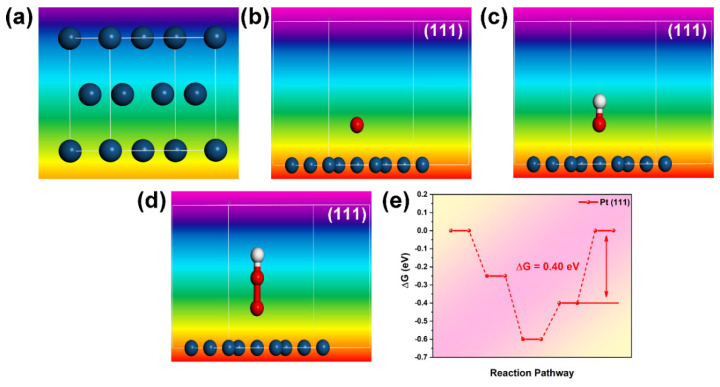
(**a**) Model image of Pt; (**b**–**d**) O*, OH*, OOH* adsorption state on (111) of Pt; (**e**) Reaction step diagram of Pt (111).

Models of Fe_3_O_4_ were designed, and the adsorption energies of intermediate states OOH*, OH*, and O* were analyzed based on the (311) crystal plane, as shown in Figure 11. The calculations were based on the GGA-PBE functional and utilized ultrasoft pseudopotentials with an energy cutoff of 500 eV. The cleavage plane chosen for analysis is (311), and a vacuum thickness of 20.0 Å is applied. The (311) crystal plane of Fe_3_O_4_ is prominent, according to XRD analysis. The active sites are located at the top positions occupied by the Fe atom. The results indicate that O*, OH*, and OOH* all exhibit adsorbed states. The calculated reaction barrier, as depicted in Figure 11e, is 0.57 eV, which is close to the adsorption barrier (0.40 eV) of Pt (111) (Figure 12e). This suggests that Fe_3_O_4_ holds theoretical potential as a substitute for Pt (111).

**Figure 13:**
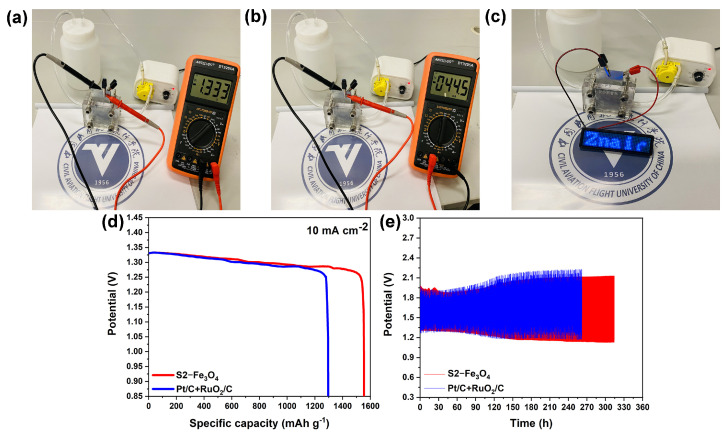
(**a**) Zn–air battery’s open circuit potential, (**b**) Current density; (**c**) Zn–air battery powers LED light, (**d**) Discharging plot, (**e**) Charge–discharging plot of S2-Fe_3_O_4_ and Pt/C + RuO_2_/C.

An investigation of the electrocatalytic performance of S2-Fe_3_O_4_ was conducted in this work by assembling a Zn–air battery. The performance of the Zn–air battery was evaluated by measuring the open circuit voltage and current density, which were found to be −1.33 V and −44.5 mA, respectively, as shown in Figure 13a,b. Notably, these results resulted in the illumination of the LED light in Figure 13c. This outcome suggests that S2-Fe_3_O_4_ exhibits promising potential as an ORR catalyst for Zn–air batteries. According to the study’s results, S2-Fe_3_O_4_ has a lot of potential for improving Zn–air battery performance, which will progress the development of efficient and renewable energy sources.

In Figure 13d, a long-term discharge test was carried out on S2-Fe_3_O_4_ with the discharge current density set at 10 mA cm^−2^. The results show that the specific capacity of S2-Fe_3_O_4_ is as high as 1513 mAh g^−1^, while that of the Pt/C + RuO₂/C mixed slurry is 1255 mAh g^−1^. As shown in Figure 13e, when a cyclic charge–discharge test was conducted at a current density of 10 mA cm^−2^, the service life of S2-Fe_3_O_4_ exceeded 310 h, while that of Pt was 260 h. Based on the above, the performance of the Zn–air battery with S2-Fe_3_O_4_ is superior to that with Pt/C + RuO_2_/C, indicating that S2-Fe_3_O_4_ has application potential.

## 4. Conclusions

In this work, high dispersion, small nanoscale, and uniform Fe_3_O_4_ nanoparticles for an efficient ORR catalyst were synthesized using FeSO_4_·7H_2_O, NaOH, and 1,4,5,8-naphthalene tetracarboxylic dianhydride as precursors, with the particle morphology controlled by adding the surfactant SDS. Increasing SDS concentration resulted in a transition from large irregular and agglomerated particles (S0-Fe_3_O_4_: 100–500 nm) to irregular particles (S1-Fe_3_O_4_: 0–450 nm), and finally to high dispersion and small nanoscale nanoparticles (S2-Fe_3_O_4_: 30–40 nm). This morphology exposes the active sites of the Fe element’s content, enhancing electrocatalytic performance. Additionally, SDS improves Fe element content, specific surface area, and pore density without altering the crystal lattice structure. Electrochemically, S2-Fe_3_O_4_ shows significant improvement compared to S0-Fe_3_O_4_, with enhanced peak potential, onset potential, half-wave potential, and limiting diffusion current density. The reaction barriers of Fe_3_O_4_ (0.57 eV) closely approach those of Pt material (0.40 eV), indicating the potential of Fe_3_O_4_ as a Pt alternative. Furthermore, S2-Fe_3_O_4_ achieves an open circuit voltage of −1.33 V in a Zn–air battery, successfully illuminating an LED light. Overall, this study demonstrates the controlled synthesis of high dispersion, small nanoscale, uniform Fe_3_O_4_ nanoparticles with promising potential as next-generation electrocatalysts, surpassing commercial Pt-based catalysts in stability and resistance to poisoning.

## Data Availability

Data, such as code, are not available in this work.

## Supplementary Materials

The following supporting information can be downloaded at: https://www.mdpi.com/article/10.3390/molecules30081753/s1, Figure S1: Repeated LSV curves of S2-Fe_3_O_4_ and S2-Fe_3_O_4_-1; Figure S2: FTIR spectroscopy of (a) S2-Fe_3_O_4_, (b) NTCDA, (c) S2-Fe_3_O_4_-B, and (d) S2-Fe_3_O_4_-B-1.
