# Supplementary material for: Nanoscale Fe3O4 Electrocatalysts for Oxygen Reduction Reaction
Source: Molecules. 2025 Apr 14;30(8):1753. doi: 10.3390/molecules30081753 (PMC12029231; doi:10.3390/molecules30081753)
Supplement: Supplementary file 1 [file molecules-30-01753-s001.zip › molecules-3498477-supplementary.pdf]

Nanoscale Fe_3O_4 Electrocatalysts for Oxygen Reduction Reaction

Junjie Zhang[1#*], Jilong Wang[1#], Yaoming Fu[1], Xing Peng[1], Maosong Xia[1], Weidong Peng[1], Yaowei Liang[2], and Wuguo Wei[1*]

[#] The contributions of the authors are the same.

[1] *Civil Aviation Flight University of China, 618000, Chengdu, China*

[2] *University of Toronto, M5S 2E8, Toronto, Canada*

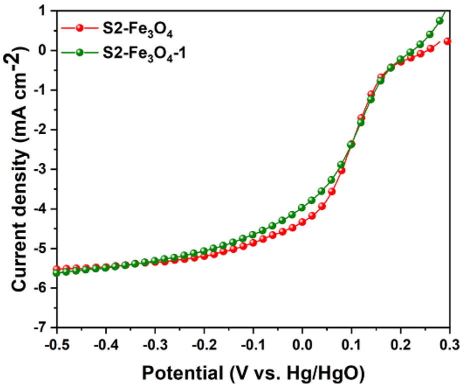

Fig. S1 Repeated LSV curves of S2-Fe_3O_4 and S2- Fe_3O_4-1

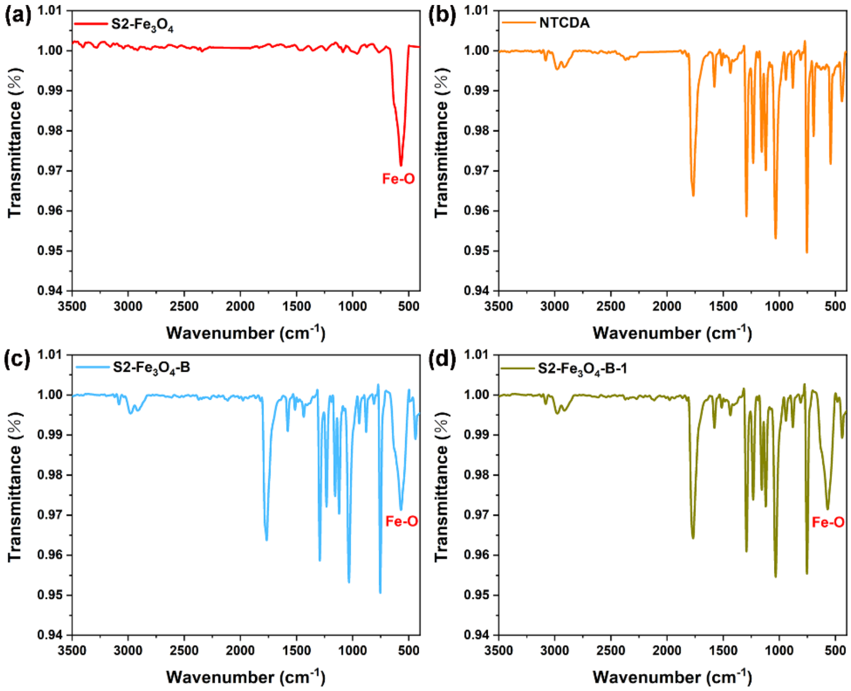

Fig. S2 FTIR spectroscopy of (a) S2-Fe_3O_4, (b) NTCDA, (c) S2-Fe_3O_4-B, and (d) S2-Fe_3O_4-B-1.
